# Psychometric properties of pain measurements for people living with dementia: a COSMIN systematic review

**DOI:** 10.1007/s41999-022-00655-z

**Published:** 2022-05-27

**Authors:** Toby O. Smith, Karmen Harvey

**Affiliations:** 1 Nuffield Department of Orthopaedics, Rheumatology and Musculoskeletal Sciences, Botnar Research Centre, NDORMS, University of Oxford, Oxford, OX3 7LD UK; 2 Faculty of Medicine and Health Sciences, University of East Anglia, Norwich, NR4 7TJ UK

**Keywords:** Pain, Distress, Outcome measure, Instrument, Older people, Cognitive impairment

## Abstract

**Purpose:**

Detecting pain in older people with dementia is challenging. Consequentially, pain is often under-reported and under-treated. There remains uncertainty over what measures should be promoted for use to assess pain in this population. The purpose of this paper is to answer this question.

**Methods:**

A search of clinical trials registered on the ClinicalTrial.gov and ISRCTN registries was performed to identify outcome measures used to assess pain in people with dementia. Following this, a systematic review of published and unpublished databases was performed to 01 November 2021 to identify papers assessing the psychometric properties of these identified measures. Each paper and measure was assessed against the COSMIN checklist. A best evidence synthesis analysis was performed to assess the level of evidence for each measure.

**Results:**

From 188 clinical trials, nine outcome measures were identified. These included: Abbey Pain Scale, ALGOPLUS, DOLOPLUS-2, Facial Action Coding System, MOBID-2, self-reported pain through the NRS or VAS/thermometer or Philadelphia Geriatric Pain Intensity Scale, PACSLAC/PACSLAC-2, Pain Assessment in Advanced Dementia (PAINAD), and Checklist for non-verbal pain behavior (CNPI). From these, 51 papers (5924 people with dementia) were identified assessing the psychometric properties of these measures. From these, there was strong- and moderate-level evidence to support the use of the facial action coding system, PACSLAC and PACSLAC-II, CNPI, DOLOPLUS-2, ALGOPLUS, MOBID, and MOBID-2 tools for the assessment of pain with people living with dementia.

**Conclusion:**

Whilst these reflect measurement tools used in research, further consideration on how these reflect clinical practice should be considered.

**PROSPERO registration:**

CRD42021282032

**Supplementary Information:**

The online version contains supplementary material available at 10.1007/s41999-022-00655-z.

## Key summary points

**Aim:**

To determine the psychometric properties of the most frequently used pain measurement tools in research of people living with dementia.

**Findings:**

There was strong and moderate level evidence to support the use of the facial action coding system, PACSLAC and PACSLAC-II, CNPI, DOLOPLUS-2, ALGOPLUS, MOBID and MOBID-2 tools for the assessment of pain with people living with dementia. There was limited evidence to support the use of the Abbey Pain Scale, PAINAD and self-reported pain through verbal rating pain score.

**Message:**

This study has identified which outcome measures are the most robust to assess pain in older people with dementia.

**Supplementary Information:**

The online version contains supplementary material available at 10.1007/s41999-022-00655-z.

## Introduction

Dementia is a major, worldwide health challenge predominantly affecting older people. It has an estimate global prevalence of 45 million people [1]. Pain is frequently reported in older people with approximately 20–50% living with chronic pain [2]. Managing pain can be difficult. There are challenges surrounding adherence and adoption of interventions such as exercise and medication taking. Detecting pain can also be difficult for people with dementia. Accordingly, pain in people with dementia is often under-detected and under-treated [3].

Self-reported pain scales such as numerical rating scales (NRS) are most frequently used to assess pain. For these patients, self-reported pain alone may not be sufficient [3]. Observed behavioural indicators of pain such as verbal complaints, sighing, moaning, agitation, crying, grimacing, rapid blinking, restlessness, rubbing, disorientation, or aggression may be valuable [4, 5].

Lichtner et al. [6] previously identified eight literature reviews reporting measurements and psychometric properties of tools assessing pain in people with dementia. No single tool was identified as more reliable and valid than others, with a wide variation in the reliability and validity. However, the search from the most recent review was performed in 2013. Furthermore, no studies have assessed the psychometric properties of outcome measures against the COnsensus-based Standards for the selection of health Measurement INstruments (COSMIN) checklist. This is a major limitation as the COSMIN checklist [7] is a robust assessment of both methodological quality of studies assessing measurement properties, with the quality of the outcome measure itself. Through this, the COSMIN checklist offers a robust, evidence-based recommendation on the quality of outcome measures selection in research and clinical practice [7].

The assessment of pain using a valid and accurate measurement is the basis for successful pain management [8]. However, there remains uncertainty on the appropriateness of these measures. Accordingly, the purpose of this systematic review was to determine the psychometric properties of the most frequently used pain measurement tools in research of people living with dementia.

## Methods

This systematic review was conducted according to the COSMIN guidance [7] and reported in accordance with the PRISMA statement [9]. The study protocol was registered prior to commencing (PROSPERO registration: CRD42021282032).

### Search strategy

Search 1: To identify the measurement tools currently used to measure pain in clinical trials of people living with dementia, we performed a search of the databases ClinicalTrial.gov and ISRCTN from inception to 01 October 2021. We used the search terms “Dementia OR cognitive impairment” AND “pain”.

Search 2: A systematic review was undertaken of published and unpublished sources to identify potentially eligible studies assessing the psychometric properties of pain measurement tools identified from Search 1. We searched the published databases: Medline, CINHAL, EMBASE, AMED, PsycINFO, and DARE from database inception to 01 November 2021. We also searched the trial registry and unpublished literature databases OpenGrey, Clinicaltrials.gov, and ISRCTN registries from inception to 01 November 2021. The search terms used for the EMBASE database are presented in Supplementary File 1. These were based on the COSMIN search filters to identify studies of psychometric properties linked to terms related to dementia, cognitive impairment, and pain. The search strategy was optimised for each electronic database search. The reference lists of all potentially eligibility studies were reviewed, and the corresponding authors from each included study were contacted and asked to review the search results.

### Eligibility assessment

For both Search 1 and 2, studies were included if they recruited people, aged 60 years and older, with dementia. Dementia criteria such as the Diagnostic and Statistical Manual of Mental Disorders, Revised Fourth Edition (DSM IV) [10], National Institute of Neurological and Communicative Disorders and Stroke/Alzheimer’s Disease and Related Disorders Association (NINCDS/ADRDA) [11], and the National Institute for Neurological Disorders and Stroke-Association Internationale pour la Recherche et Enseignement en Neurosciences (NINCDS-AIREN) [12] were considered appropriate. Where self-reported dementia was reported, further scrutiny of the characteristics of the population in relation to severity of cognitive impairment, age, and comorbidities were considered. Where uncertain, corresponding authors were asked to verify the approach used to define dementia. All stages and severities of dementia were eligibility, i.e., mild, moderate, and severe. Whilst it is acknowledged that pain assessment tools have been developed for other, non-dementia, patient groups with cognitive impairment [13], these were excluded from this review unless there was sufficient evidence that participants presented with dementia.

We did not restrict the form, cause, or pathology causing pain. Through this, participant’s pain arise from musculoskeletal, post-surgery, medical, and cancer-related sources.

We included studies regardless of setting, i.e., acute, community, residential, or nursing home. We excluded studies not published in English, narrative, and systematic reviews, although reviewed the reference lists of these publications to identify any previously omitted studies.

For Search 2, we included all full-text publications which reported any assessment of the psychometric properties of measurement tools identified from Search 1. Papers which included findings on pain management were considered if they also provided data on the psychometric properties of a pain measurement tool. We only included studies which reported one or more of the COSMIN taxonomy of: internal consistency, test–retest reliability, measurement error, content validity, structural validity, construct validity/hypotheses testing, cross-cultural validity, criterion validity, or responsiveness [7].

### Study identification

The search results were screened against the eligibility criteria by two reviewers (TS, KH). This was initially by title and abstract, and then by full-text version. Screening was performed by each reviewer independently. When consensus on study eligibility could not be reached, agreement was reached through discussion.

### Data extraction

For each included study, data were extracted independently by one reviewer (TS). This was then verified for accuracy by a second reviewer (KH). Where disagreements occurred, these were resolved through discussion.

Data were extracted onto a bespoke data extraction table. Data extracted included: measurement tool name, setting tested, country of assessment, method of administration, person administered, duration between testing (if appropriate), patient participant characteristics (number and response rate), age, gender, diagnosis of pain, diagnosis of dementia, severity of dementia), and psychometric outcomes (reliability, validity, and responsiveness).

### Risk of bias

To assess the methodological quality of the included studies, the Consensus-based Standards for the selection of health Measurement Instruments (COSMIN) checklist [14] was used. The COSMIN checklist assesses the following measurement properties: content validity, structural validity, internal consistency, cross-cultural validity/measurement invariance, reliability, measurement error, criterion validity, hypotheses testing for construct validity, and responsiveness. The overall quality of how each measurement property was evaluated on a four-point scale: very good, adequate, doubtful, or inadequate, as per the COSMIN guidance. The methodological quality score per property was then obtained by taking the lowest rating of any item in each box—worst score counts principle. Two reviewers (TS, KH) assessed each study using this approach independently with disagreements resolved through consensus.

### Data analysis

The psychometric properties of each measurement tool were reported narratively. Through this descriptive statistics, inferential statistics and degrees of variance were reported from included studies. Analysis was made following Chiarotto et al. [15] best evidence synthesis approach where ‘strong’ was a measurement tool which demonstrate consistent findings in multiple studies of good methodological quality OR in one study of excellent methodological quality; ‘moderate’ demonstrated consistent findings in multiple studies of fair methodological quality OR in one study of good methodological quality, ‘limited’ demonstrated on study of fair methodological quality, conflicting demonstrated conflicting findings and ‘unknown’ was only for studies of poor methodological quality or no studies reporting a measure.

## Results

### Search 1: identification of measurement tools

In total, 188 individual clinical trials were identified from Search 1. Of these, 56 were identified which reported measuring pain with participants living with dementia. A summary of these studies is presented in Table 1.

**Table 1:** Summary of trial registers which reported measuring pain in people with dementia

		Frequency	%
*N*		56	100
Date study commenced	2007–2011	2	3.6
	2012–2016	17	30.4
	2017–2021	37	66.0
Country of origin	Australia	1	1.8
	Belgium	1	1.8
	Canada	6	10.7
	China	1	1.8
	France	7	12.5
	Germany	1	1.8
	Italy	2	3.6
	Netherlands	2	3.6
	Norway	7	12.5
	Spain	2	3.6
	Switzerland	1	1.8
	Taiwan	3	5.4
	UK	3	5.4
	USA	19	33.9
Type of intention	Pharmacology agent	13	23.2
	Non-pharmacology intervention	43	76.8
Mean *N* (SD)		268.2 (576.1)	
Participant degree of cogitative impairment	Mild	11	19.6
	Mild–moderate	10	17.9
	Mild–severe	14	25.0
	Moderate–severe	14	25.0
	Severe	7	12.5
Setting	Hospital	9	16.1
	Community-dwelling	21	37.5
	Care home	22	39.3
	Not stated	4	7.1
Mean follow-up period (SD)		26.2 (25.9)	
Pain measure	Abbey pain scale	2	3.6
	ALGOPLUS	1	1.8
	Brief pain inventory	1	1.8
	Comfort assessment in dying with dementia	1	1.8
	DOLOPLUS-2	1	1.8
	Edmonton symptom assessment scale	2	3.6
	EQ-5D	5	8.9
	Facial action coding system	1	1.8
	GLOBAL PROMIS-10	1	1.8
	McGill pain map	1	1.8
	SF-36	3	5.4
	Medication use	2	3.6
	MOBID-2	9	16.1
	Self-reported (NRS/VAS pain/verbal descriptor scale/thermometer)	9	16.1
	PACSLAC and PACSLAC-2	6	10.7
	Pain assessment in advanced dementia (PAINAD)	9	16.1
	Philadelphia geriatric pain intensity scale patient and caregiver responded	2	3.6
	Resident assessment index-minimum dataset	2	3.6
	Symptom Management—end of life for dementia	1	1.8
	WOMAC	1	1.8
	Checklist for non-verbal pain behavior	1	1.8

*SD* standard deviation

From the list generated from Search 1, we excluded all measures which did not specifically assess pain but included pain as a sub-domain of an instrument, e.g., SF-36, WOMAC, and EQ-5D. From this, seven outcomes were excluded (Comfort Assessment in Dying with Dementia, Edmonton Symptom Assessment Scale, EQ-5D, GLOBAL PROMIS-10, SF-36, Resident Assessment Index-Minimum Dataset, and Symptom Management—End of Life for Dementia). We excluded measurement tools which were not designed for people with cognitive impairment. Accordingly, three instruments were excluded (Brief Pain Inventory, McGill Pain Map, and WOMAC). Resultantly, the psychometric properties of nine measurement tools formed the basis of Search 2 (Abbey Pain Scale, ALGOPLUS, DOLOPLUS-2, Facial Action Coding System, MOBID-2, self-reported pain through the NRS or VAS/thermometer or Philadelphia Geriatric Pain Intensity Scale, PACSLAC/PACSLAC-2, Pain Assessment in Advanced Dementia (PAINAD), and Checklist for non-verbal pain behavior (CNPI) (Supplementary File 2).

### Search 2: Psychometric tools analysis

**Fig. 1:**
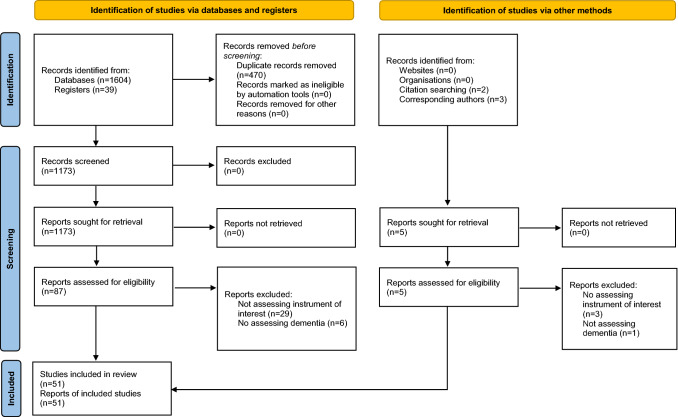
PRISMA flowchart reporting search results for Search 2

A summary of the Search 2 results is presented in Fig. 1. In total, 1173 individual citations were identified. Fifty-one studies reported data on the psychometric properties of one or more of the nine measurement tools identified in Search 1. These studies were included in the analysis.

### Characteristics of included studies and quality assessment

A summary of the characteristics of the included studies is presented in Table 2. In total, 5924 people with dementia were assessed. Mean age of population ranged from 72.5 years [16] to 87.9 years [17]. Thirteen studies were performed in a hospital setting [16, 18–29], 33 in care home facilities [17, 30–61] and two studies were based in both care home and people’s home settings [62, 63]. Two studies were performed both in care home and hospital settings [64, 65]. The location of study was not stated in Lorenzet et al. [66]. Studies were reported in 21 countries, most frequently Norway (*n* = 8) [32, 41, 48, 56–59, 63], USA (*n* = 7) [19, 33, 34, 42, 44, 60, 61], Canada (*n* = 4) [31, 52, 54, 55], and Brazil (*n* = 4)] 17,22,23,66].

**Table 2:** Summary of included studies

Study	Setting (hospital; home; care home)	Language	Severity of CI (mean)	Cohort characteristics				Country of origin	Study funding source	Measurement of pain
				*N* CI	Age (mean)	Gender	Cognitive diagnosis			
Abbey [35]	Care facility	English	Moderate to severe	61	Median: 83	40F/21 M	Dementia	Australia	JH and JD Gunn Medical Research Foundation	Abbey Pain Scale
Akbarzadeh [50]	Care facility	Swedish	Not stated	48	> 65	N/S	Dementia	Sweden	None declared	DOLOPLUS-2
Ando [25]	Hospital	Japanese	MMSE: 10	9	80	4F/5 M	Dementia	Japan	None declared	DOLOPLUS-2
Ando [26]	Hospital	Japanese	MMSE: 10.9	19	84.5	15F/4 M	Dementia	Japan	Okochi Fund at Yokufukai Geriatric Hospital	DOLOPLUS-2
Atee [39]	Care facility	English	PASCI score: 19.7	34	85.5	20F/14 M	Dementia	Australia	Alzheimer’s Australia Dementia Research Foundation	Abbey Pain Scale
Babicova [38]	Care facility	English	Moderate to severe Mean: 5.8	22	84.7	17F/5 M	Dementia	UK	None declared	Abbey Pain Scale
Batalha [21]	Hospital	Portuguese	Not stated	99	82	68F/31 M	Dementia	Portugal	None declared	PAINAD-P
Browne [30]	Care facility	English	CPS: 3.74	48	78.8	34F/19 M	Dementia	Canada	AGE WELL Network of Centres of Excellence and the Canadian Institutes of Health Research	Facial Action Coding System PACSLAC-II
Büyükturan [16]	Hospital	Turkish	MMSE: 2.15	106	72.5	54F/52 M	Dementia	Turkey	None declared	PAINAD-TR
Cantón-Habas [24]	Hospital	Spanish	GDS: 5–7	100	83.8	22F/78 M	Dementia	Spain	Junta de Andalucía	PAINAD-S
Cantón-Habas [65]	Hospital and Care facility	Spanish	GDS: 5–7	75	84.4	59F/16 M	Dementia	Spain	Junta de Andalucía	PAINAD-S
Chan [55]	Care facility	English	MMSE: 5.35	124	83.9	88F/36 M	Dementia	Canada	Alzheimer Society of Canada; Saskatchewan Health Research Foundation; University of Regina	PACSLAC-II
Chen [49]	Care facility	Chinese	MMSE: 7.46	304	79.9	129F/175 M	Dementia	Taiwan	National Science Council, Taiwan	DOLOPLUS-2
Chen [51]	Care facility	Chinese	MMSE: 5.26	241	79.2	118F/123 M	Dementia	Taiwan	National Science Council, Taiwan	DOLOPLUS-2
Cheung [53]	Care facility	English	MMSE:7.5	50	82.9	36F/14 M	Dementia	New Zealand	None declared	PACSLAC
Costardi [20]	Hospital	Italian	MMSE: 16.4	20	82	16F/4 M	Dementia	Italy	None declared	PAINAD-Italian
Ersek [44]	Care facility	English	CPS: 3.9	60	89.0	53F/7 M	Dementia	USA	National Institute of Nursing Research, USA	CNPI; PAINAD
Ersek [42]	Care facility	English	Severe	326	83.2	225F/101 M	Dementia	USA	National Institute of Nursing Research, USA	Iowa Pain Thermometer; CNPI
Feldt [19]	Hospital	English	MMSE: CI 12.2 nCI: 27.2	88	83.2	76F/12 M	Dementia	USA	University of Minnesota, USA	CNPI
Fuchs-Lacelle [52]	Care facility	English	PFQ: 44.6	40	83.2	29F/11 M	Dementia	Canada	Saskatchewan Health Research Foundation; Canadian Institutes of Health Research Career Investigator Award	PACSLAC
Hadjistavropoulos [31]	Care facility	English	CPS: 3.74	48	82.5	69F/36 M	Dementia	Canada	AGE WELL Network of Centres of Excellence and the Canadian Institutes of Health Research	Facial Action Coding System PACSLAC-II
Herr [60]	Care facility	English	Moderate-severe	138	84	63F/75 M	Dementia	USA	Department of Veterans Affairs, USA	MOBID
Holen [32]	Care facility	Norwegian	MMSE Median: 9	59	Median: 82	47F/12 M	Dementia	Norway	The Research Council of Norway	DOLOPLUS-2
Holen [63]	Care facility and hospital	Norwegian	Median MMSE: 10	73	84	54F/19 M	Dementia	Norway	The Research Council of Norway	DOLOPLUS-2
Husebo [56]	Care facility	Norwegian	MMSE: 4.3	26	87.0	23F/3 M	Dementia	Norway	The Research Council of Norway; Kavli’s Research Center for Dementia	MOBID
Husebo [58]	Care facility	Norwegian	MMSE: 4.3	26	87.0	23F/3 M	Dementia	Norway	The Research Council of Norway; Kavli’s Research Center for Dementia	MOBID
Husebo [57]	Care facility	Norwegian	MMSE: 2.4	77	84.1	61F/16 M	Dementia	Norway	The Research Council of Norway; Kavli’s Research Center for Dementia	MOBID-2
Husebo [59]	Care facility	Norwegian	MMSE: 8.1	203	85.4	149F/54 M	Dementia	Norway	The Research Council of Norway; Kavli’s Research Center for Dementia	MOBID-2
Kaasalainen [54]	Care facility	English	Not stated	338	82.8	216F/122 M	Dementia	Canada	Canadian Institutes of Health Research	PACSLAC
Kunz [18]	Hospital	German	MMSE CI: 16.3 Healthy: 29.5	42	76.7	22F/20 M	Dementia	Germany	Deutsche Forschungsgemeinschaft	Facial Action Coding System
Lautenbacher [64]	Hospital and Care facility	German	MMSE CI: 17.0 Healthy: 29.1	40	> 65	N/S	Dementia	Germany	European Cooperation in the field of Scientific and Technical Research program; Oberfranken-Stiftung	Facial Action Coding System using the PAIC-FACE-SCALE
Leong [45]	Care facility	Chinese	CPS: 3.9	88	79.6	54F/34 M	Dementia	Singapore	Tan Tock Seng Hospital	Self-reported pain, PAINAD
Lin [47]	Care facility	Chinese	MMSE: 3.20	61	76.3	29F/32 M	Dementia	China	National Science Council, Taiwan	PAINAD-C
Liu [40]	Care facility	Chinese	MMSE: CI: 9.97 nCI: 22.71	124	87.1	120F/4 M	Dementia	Hong Kong	None declared	PAINAD, PACSLAC, Abbey Pain Scale
Lorenzet [66]	Not stated	Portuguese	Not stated	N/S	N/S	N/S	Not stated	Brazil	None declared	PACSLAC
Neville [43]	Care facility	English	Moderate to severe	126	85.2	104F/22 M	Dementia	Australia	University of Queensland	Abbey Pain Scale; DOLOPLUS 2; CNPI
Nygaard [41]	Care facility	Norwegian	SPMQ: 46 missing 2 answers	46	84.7	29F/17 M	Dementia (89%)	Norway	Lions Foundation	CNPI
Parmelee [33]	Care facility	English	386 mild–severe CI	758	83.3	531F/227 M	Dementia	USA	None declared	Self-Reported Pain and Pain Thermometer
Pateux [28]	Hospital	French	MMSE: 18.0	180	83.7	133F/47 M	Dementia	France	University Hospital of Geneva	Verbal rating scale; DOLOPLUS-2
Pautex [27]	Hospital	French	MMSE: 17.8	160	85.5	114F/46 M	Dementia	France	University Hospital of Geneva	Verbal rating scale; Faces Pain Scale
Pinto [23]	Hospital	Portuguese	N/S	66	Median: 87	44F/22 M	Dementia	Brazil	None declared	PAINAD-Br
Rat [29]	Hospital	French	N/S	349	81.6	214F/135 M	Dementia	France	CNP Foundation; Laboratories Grünenthal France	Algoplus
Sefcik [61]	Care facility	English	N/S	197	84	95F/102 M	Dementia	USA	None declared	MOBID
Takai [36]	Care facility	Japanese	MMSE: 9.1	171	85.4	142F/29 M	Dementia	Japan	Kinuko Takasaki Gerontological Nursing Grant	Abbey Pain Scale-Japanese
Thé [17]	Care facility	Portuguese	N/S	50	87.8	39F/11 M	Dementia	Brazil	None declared	PACSLAC
Torvik [48]	Care facility	Norwegian	MMSE: 0	77	86	58F/19 M	Dementia	Norway	None declared	DOLOPLUS-2
Valera [22]	Hospital	Portuguese	N/S	27	81.8	19F/8 M	Dementia	Brazil	São Paulo—FAPESP; Brazilian National Council of Scientific and Technological Development—CNPq	PAINAD-Br
Van Iersel [37]	Care facility	Dutch	N/S	157	85	122F/35 M	N/S	Belgium	None declared	Abbey Pain Scale-Dutch; PAINAD-Dutch
Weiner [34]	Care facility	English	N/S	115	Median: 81	51F/64 M	Dementia	USA	National Institute of Health, USA; Arthritis Foundation, USA	Self-Reported Pain and Pain Thermometer
Zare [62]	Care facility and home	Persian	Mild–severe	100	87.3	71F/29 M	Dementia	Iran	Kashan University of Medical Sciences	P-DOLOPLUS-2; PACSLAC-2-IR
Zwakhalen [46]	Care facility	Dutch	Mild–severe	128	82.4	100F/28 M	Dementia	Netherlands	The Netherlands Organization for Scientific Research	PAINAD, PACSLAC, DOLOPLUS-2

*CI* cognitively impaired; *CNPI* checklist of nonverbal pain indicators; *CPS* cognitive performance scale; *F* female; *GDS* global deterioration score; *M* male; *MMSE* mini-mental state examination; *N/S* not stated; *nCI* not cognitively impaired; *PASCI* psychogeriatric assessment scale cognitive impairment; *SPMQ* short-portable mental status questionnaire

A summary of the findings from the COSMIN assessment is presented in Supplementary File 3. The results for the psychometric analysis are presented in Supplementary File 4. A summary of findings for the best evidence synthesis is presented as Table 3.

**Table 3:**
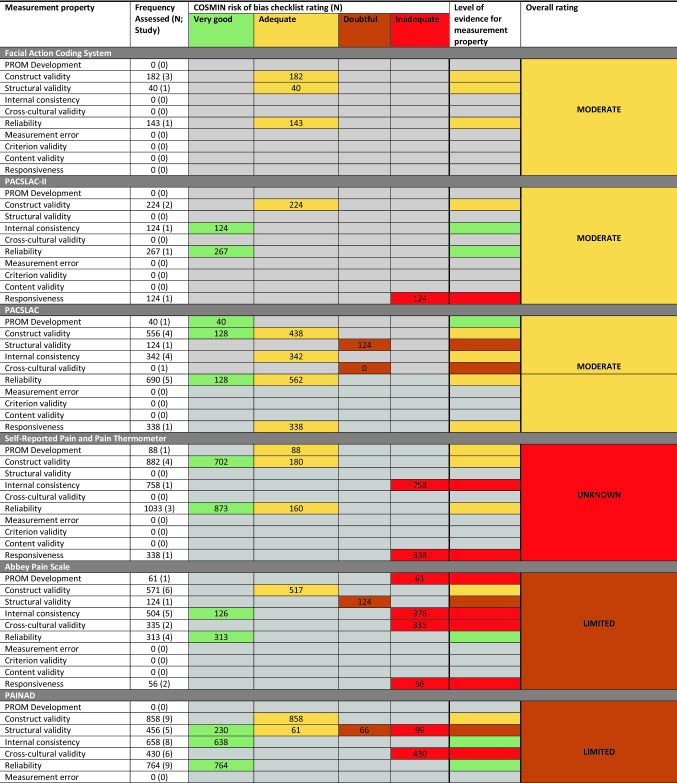

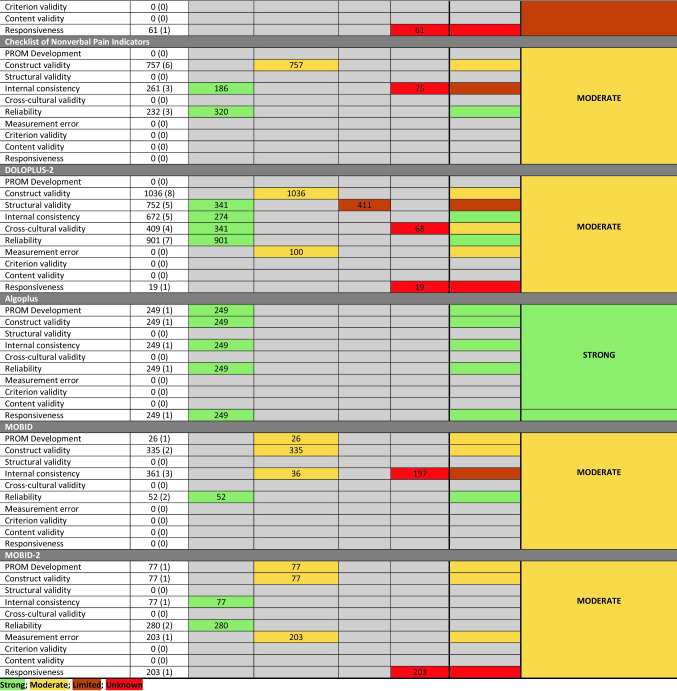
Best evidence synthesis of outcome measures used to assess pain in people with dementia against the COSMIN risk of bias checklist rating and level of evidence for the measurement property

### Abbey pain scale

Eight studies reported data on the psychometric properties of the Abbey Pain Scale [35–40, 43, 46]. Overall, there was limited evidence for the use of the Abbey Pain Scale (Table 3). There was inadequate evidence on PROM development, internal consistency (Cronbach: 0.65–0.74), cross-cultural validity, and responsiveness (*p* < 0.001). There was adequate evidence for the assessment of construct validity (*R* = 0.49–0.91) and very good evidence for reliability (inter-rater: 0.75–0.88; intra-rater: 0.66–0.68). The level of evidence for structural validity was doubtful (Cronbach: 0.76).

### Pain assessment in advanced dementia (PAINAD)

Twelve studies assessed the PAINAD [16, 20–24, 40, 44–47, 65]. Overall, the level of evidence for the PAINAD tool was limited (Table 3). Whilst there was an adequate level of evidence for construct validity (*R* = 0.48–0.88), very good level of evidence for internal consistency (Cronbach alpha: 0.65–0.84), and reliability (intra-rater: 0.71–0.89; inter-rater: 0.79–0.94), there was inadequate evidence for cross-cultural validity and responsiveness (*p* < 0.001). There was doubtful level of evidence for structural validity (variance explained: 46.5–68.9%).

### Facial action coding system

Five studies provided data on the facial action coding system [18, 27, 30, 31, 64]. These demonstrated moderate evidence for the use of this measurement tool (Table 3). There was adequate evidence for construct validity (*R* = 0.116–0.463), structural validity (*p* = 0.06 to *p* < 0.001), and reliability (inter-rater: 0.94).

### Checklist for non-verbal pain behavior (CNPI)

Six studies presented data on the psychometric properties of the CNPI [19, 41–44, 55]. Overall, there was moderate evidence for the CNPI (Table 3). There was adequate evidence for construct validity (*R* = 0.46–0.88) and very good evidence of reliability (intra-rater: 0.23–0.65; inter-rater: 0.45–0.59). However, there was inadequate evidence for internal consistency (Cronbach alpha: 0.64–0.90).

### Self-reported pain through verbal rating pain score

Ten studies assessed the psychometric properties of self-reported/verbal rating pain measures [27–29, 33–35, 42, 45, 51, 54]. Overall, there was limited evidence supporting the use of these tools (Table 3). Whilst there was adequate evidence on PROM development, construct validity (*R* = 0.30–0.95), and reliability (intra-rater: 0.71–0.84; inter-rater: 0.81–0.97), there was inadequate evidence on internal consistency (Cronbach: 0.74–0.84) and responsiveness (*p* = 0.03).

### ALGOPLUS

One study, performed in a French hospital setting, presented data on the psychometric properties of the ALGOPLUS instrument [29]. This provided strong evidence for this tool (Table 3). Data reported very high construct validity (*r*^2^ = 0.81; *p* < 0.001), very high inter-rater reliability (0.812), and internal validity (KR-20: 0.712) and responsiveness to treatment (*p* < 0.001).

### MOBID and MOBID-2

Four studies presented data on the psychometric properties of the MOBID [56, 58, 60, 61]. Overall, the MOBID instruments demonstrated moderate evidence (Table 3). If offered adequate evidence for PROM development and construct validity (*R* = 0.51–0.54 [60, 61]. Whilst the instrument demonstrated doubtful evidence for internal consistency, the values were high (Cronbach: 0.83–0.89), and it demonstrated adequate evidence for reliability (inter-rater: 0.86–0.97; intra-rater: 0.79–0.92).

Two studies reported data on the MOBID-2 [57, 59] instrument. It demonstrated moderate evidence for use (Table 3). There was adequate evidence for PROM development and construct validity (*R* = 0.61), and measurement error (Standard Error of Measurement (SEM): 1.4). Whilst there was inadequate evidence for the responsiveness, the minimally clinically important difference (MCID) was reported as three points and reported to be responsive to treatment (*p* < 0.001). There was very good evidence for the MOBID-2 for internal consistency (Cronbach: 0.82–0.84) and reliability (inter-rater: 0.94; intra-rater: 0.85–0.92).

### PACSLAC and PACSLAC-II

Four studies assessed the PACSLAC-II [30, 31, 55, 62]. They suggested moderate evidence to support the use of this measurement tool (Table 3). There was very good evidence for internal consistency (Cronbach: 0.74–0.77), and reliability (inter-rater: 0.63–0.86) and adequate evidence for construct validity (*R* = 0.54–0.68). However, there was inadequate evidence for the assessment of responsiveness (*p* < 0.01).

The PACLAC was assessed in six studies [17, 40, 52–54, 66]. This demonstrated moderate evidence (Table 3). There was very good evidence for PROM development. There was adequate evidence for construct validity (*R* = 0.54–0.72), internal consistency (Cronbach alpha: 0.77–0.87), reliability (inter-rater: 0.52–0.96; intra-rater: 0.86), and responsiveness (*p* < 0.001). There was doubtful evidence for structural validity and cross-cultural validity.

### DOLOPLUS-2

Thirteen studies assessed the psychometric properties of the DOLOPLUS-2 [25–28, 32, 44, 46, 48–51, 62, 63]. Overall, there was moderate evidence to support the use of this measurement tool. It demonstrated very good evidence for the assessment of internal consistency (Cronbach: 0.770–0.95) and reliability (intra-rater: 0.71; inter-rater: 0.35–0.86). There was adequate evidence for construct validity (*R* = 0.33–0.70), measurement error (SEM: ± 1.759), and cross-cultural validity. There was doubtful evidence for structural validity (explained variance: 36.9–76.1%) and inadequate evidence on responsiveness (*p* < 0.001).

## Discussion

The findings indicate strong and moderate evidence to support the use of the facial action coding system, PACSLAC and PACSLAC-II, CNPI, DOLOPLUS-2, ALGOPLUS, MOBID, and MOBID-2 tools. There is limited evidence for the Abbey Pain Scale, self-reported pain measures, and the PAINAD tool.

The literature highlights the challenges of assessing pain with people living with dementia [3, 4, 67]. Challenges have included insufficient time to use measurement tools [68, 69], user’s uncertainty over the reliability of these [70], access to physically finding and using the measurement tools [71], and perceived superiority of observational methods of behaviors and physical manifestations of pain [70]. Whilst there is a bias to observational manifestation in a number of the supported measurement tools recommended, the time to complete and interpret these may act as a further barrier to adoption. Consideration of such potential challenges may be made when exploring the implementation of recommended measurement tools.

Under-treatment of pain in people with dementia has been attributed to challenges in recognition and assessment of pain, coupled with reservations on polypharmacy and side effects of analgesia [72]. Achterberg et al. [73] highlighted the frequently seen scenario where people with dementia are prescribed analgesics, but due to concerns around side effects, particularly regarding non-steroidal anti-inflammatory drugs, opioids, and adjunct analgesics, the medications are either not administer or are at a sufficient dosage to manage symptoms. This was clearly illustrated in Roitto et al.’s [74] survey where although 19% of their 327 cohort of people living in nursing homes with dementia were prescribed opioids, 79% were still in pain. Whilst this study has highlighted potentially robust pain measurement tools for this population, implementing both the assessment and subsequent treatment to improve pain management is required.

Pain assessment ideally considers several pain dimensions. These include: intensity, location, affect, cognition, behavior, and social accompaniments [72]. Measurement tools, most notably the DOLOPLUS-2, are multi-dimensional. Conversely, self-reported VAS/NRS of observation are unidimensional. However, it is acknowledged that assessment of some dimensions, notably pain cognition, can be more challenging due to communication and cognitive barriers. Focusing on single dimensions should be avoided to negate the risks of under-reporting/under-representing pain experienced by individuals.

Whilst reliability and construct validity were well explored, there remains limited evidence of the responsiveness, structural validity, and measurement error for many of the identified measures. This may be a reason for why pain measurement tools are poorly adopted into practice. Improving confidence around how measurement tools are used and interpreted may promote the implementation of such tools. Furthermore, as observational tools were most widely assessed, understanding the ‘normal’ or familiar behaviors of a person with dementia is important to recognise when something abnormal or noxious is being felt. No studies assessed the difference in reliability or validity when the assessment was performed by a healthcare professional versus a close relative or friend who may be more familiar with the individual. This may be an important area for future study, particularly when considering the adoption of pain assessment instruments in community and non-health or social care profession settings.

This systematic review presents with a number of strengths and limitations. A major strength is the adoption of the COSMIN evaluation. This approach ensured that the reader could be fully informed on the confidence with the recommendations made based on the evidence. Three important limitations should be considered. First, a comprehensive approach to reporting the psychometric properties of the most frequently used measurement instruments in research was adopted to aid prioritisation. However, this meant measurement tools used in clinical practice but not trials may have been omitted. Second, given the methods adopted through Search 1 to identify potential measurement tools, more recent tools such as the ePAT were not included in the analysis [39]. Consideration of this and inclusion of forthcoming evidence on psychometric properties should be made to update the findings as new evidence evolves in the field. Second, there was insufficient evidence to assess differences in recommendations based on severity of dementia. Evaluation on the impact of severity of cognitive impairment on the performance of the identified measurement tools would be warranted. Finally, there were challenges cause by poor reporting within included studies. There was insufficient detail within included studies to ascertain whether pain assessment instruments assessed acute or chronic pain, or whether individuals were taking analgesia or not. This may impact on the generalisability of the findings into practice and should be consider when reporting future studies in this area.

To conclude, there is strong and moderate evidence to support the use of the facial action coding system, PACSLAC and PACSLAC-II, CNPI, DOLOPLUS-2, ALGOPLUS, MOBID, and MOBID-2 tools for the assessment of pain with people living with dementia. Whilst these reflect measurement tools used in research, further consideration on how these reflect clinical practice, and lessons on how to implement these tools into practice should be considered to improve the detection and management of pain for people with dementia.

## Supplementary Information

Below is the link to the electronic supplementary material.

Supplementary file1 (DOCX 38 KB)

## References

[1] Prince M, Ali GC, Guerchet M, Prina AM, Albanese E, Wu YT (2016). Recent global trends in the prevalence and incidence of dementia, and survival with dementia. Alzheimers Res Ther.

[2] Apinis C, Tousignant M, Arcand M, Tousignant-Laflamme Y (2014). Can adding a standardized observational tool to interdisciplinary evaluation enhance the detection of pain in older adults with cognitive impairments?. Pain Med.

[3] Zwakhalen SM, Hamers JP, Abu-Saad HH, Berger MP (2006). Pain in elderly people with severe dementia: a systematic review of behavioural pain assessment tools. BMC Geriatr.

[4] Malara A, De Biase GA, Bettarini F, Ceravolo F, Di Cello S, Garo M, Praino F, Settembrini V, Sgrò G, Spadea F, Rispoli V (2016). Pain Assessment in elderly with behavioral and psychological symptoms of dementia. J Alzheimers Dis.

[5] American Geriatrics Society (2009). AGS panel on the pharmacological management of persistent pain in older persons. J Am Geriatr Soc.

[6] Lichtner V, Dowding D, Esterhuizen P, Closs SJ, Long AF, Corbett A, Briggs M (2014). Pain assessment for people with dementia: a systematic review of systematic reviews of pain assessment tools. BMC Geriatr.

[7] Prinsen CAC, Mokkink LB, Bouter LM, Alonso J, Patrick DL, de Vet HCW, Terwee CB (2018). COSMIN guideline for systematic reviews of patient-reported outcome measures. Qual Life Res.

[8] Corbett A, Husebo B, Malcangio M, Staniland A, Cohen-Mansfield J, Aarsland D, Ballard C (2012). Assessment and treatment of pain in people with dementia. Nat Rev Neurol.

[9] Page MJ, McKenzie JE, Bossuyt PM, Boutron I, Hoffmann TC, Mulrow CD, Shamseer L, Tetzlaff JM, Akl EA, Brennan SE, Chou R, Glanville J, Grimshaw JM, Hróbjartsson A, Lalu MM, Li T, Loder EW, Mayo-Wilson E, McDonald S, McGuinness LA, Stewart LA, Thomas J, Tricco AC, Welch VA, Whiting P, Moher D (2021). The PRISMA 2020 statement: an updated guideline for reporting systematic reviews. BMJ.

[10] American Psychiatric Association (2000). Diagnostic and statistical manual of mental disorders, fourth edition, text revision (DSM-IV-TR).

[11] McKhann G, Drachman D, Folstein M, Katzman R, Price D, Stadlan EM (1984). Clinical diagnosis of Alzheimer’s disease: report of the NINCDS-ADRDA Work Group under the auspices of department of health and human services task force on Alzheimer’s disease. Neurology.

[12] Roman GC, Tatemichi TK, Erkinjuntti T, Cummings JL, Masdeu JC, Garcia JH, Amaducci L, Orgogozo JM, Brun A, Hofman A (1993). Vascular dementia: diagnostic criteria for research studies. Report of the NINDS-AIREN International Workshop. Neurology.

[13] Decker SA, Perry AG (2003). The development and testing of the PATCOA to assess pain in confused older adults. Pain Manag Nurs.

[14] Mokkink LB, Terwee CB, Knol DL, Stratford PW, Alonso J, Patrick DL, Bouter LM, de Vet HC (2010). The COSMIN checklist for evaluating the methodological quality of studies on measurement properties: a clarification of its content. BMC Med Res Methodol.

[15] Chiarotto A, Maxwell LJ, Terwee CB, Wells GA, Tugwell P, Ostelo RW (2016). Roland-Morris disability questionnaire and Oswestry disability index: which has better measurement properties for measuring physical functioning in nonspecific low back pain? Systematic review and meta-analysis. Phys Ther.

[16] Büyükturan O, Lkin Naharci M, Büyükturan B, Kirdi N, Yetiş A (2018). The Turkish version of pain assessment in advanced dementia (PAINAD) scale. Noro Psikiyatr Ars.

[17] Thé KB, Gazoni FM, Cherpak GL, Lorenzet IC, Alves dos Santos L, Nardes EM, dos Santos FC (2016). Pain assessment in elderly with dementia: Brazilian validation of the PACSLAC scale. Einstein.

[18] Kunz M, Scharmann S, Hemmeter U, Schepelmann K, Lautenbacher S (2007). The facial expression of pain in patients with dementia. Pain.

[19] Feldt KS (2000). The checklist of nonverbal pain indicators (CNPI). Pain Manag Nurs.

[20] Costardi D, Rozzini L, Costanzi C, Ghianda D, Franzoni S, Padovani A, Trabucchi M (2007). The Italian version of the pain assessment in advanced dementia (PAINAD) scale. Arch Gerontol Geriatr.

[21] Batalha LMC; Duarte CIA, do Rosário RAF, da Costa MFSP, Pereira VJR, Morgado TMM. Adaptação cultural e propriedades psicométricas da versão portuguesa da escala Pain Assessment in Advanced Dementia. Rev Esc Enferm USP. 2012;8:7–16.

[22] Valera GG, Carezzato NL, Vale FA, Hortense P (2014). Cultural adaptation of the scale pain assessment in advanced dementia—PAINAD to Brazil. Rev Esc Enferm USP.

[23] Pinto MC, Minson FP, Lopes AC, Laselva CR (2015). Cultural adaptation and reproducibility validation of the Brazilian Portuguese version of the pain assessment in advanced dementia (PAINAD-Brazil) scale in non-verbal adult patients. Einstein.

[24] Cantón-Habas V, Carrera-González MDP, Moreno-Casbas MT, Rich-Ruiz M (2021). Spanish adaptation and validation of the pain assessment scale in advanced dementia (PAINAD) in patients with dementia and impaired verbal communication: cross-sectional study. BMJ Open.

[25] Ando C, Hishinuma M (2010). Development of the Japanese DOLOPLUS-2: a pain assessment scale for the elderly with Alzheimer’s disease. Psychogeriatrics.

[26] Ando C, Ito Y, Amemiya S, Tamura K, Kako K, Tsuzura S, Yoshida R, Hishinuma M (2016). Effectiveness of the Japanese DOLOPLUS-2: a pain assessment scale for patients with moderate-to-severe dementia. Psychogeriatrics.

[27] Pautex S, Herrmann F, Le Lous P, Fabjan M, Michel JP, Gold G (2005). Feasibility and reliability of four pain self-assessment scales and correlation with an observational rating scale in hospitalized elderly demented patients. J Gerontol A Biol Sci Med Sci.

[28] Pautex S, Herrmann FR, Michon A, Giannakopoulos P, Gold G (2007). Psychometric properties of the Doloplus-2 observational pain assessment scale and comparison to self-assessment in hospitalized elderly. Clin J Pain.

[29] Rat P, Jouve E, Pickering G, Donnarel L, Nguyen L, Michel M, Capriz-Ribière F, Lefebvre-Chapiro S, Gauquelin F, Bonin-Guillaume S (2011). Validation of an acute pain-behavior scale for older persons with inability to communicate verbally: Algoplus. Eur J Pain.

[30] Erin Browne M, Hadjistavropoulos T, Prkachin K, Ashraf A, Taati B (2019). Pain expressions in dementia: validity of observers’ pain judgments as a function of angle of observation. J Nonverbal Behav.

[31] Hadjistavropoulos T, Browne ME, Prkachin KM, Taati B, Ashraf A, Mihailidis A (2018). Pain in severe dementia: a comparison of a fine-grained assessment approach to an observational checklist designed for clinical settings. Eur J Pain.

[32] Hølen JC, Saltvedt I, Fayers PM, Bjørnnes M, Stenseth G, Hval B, Filbet M, Loge JH, Kaasa S (2005). The Norwegian Doloplus-2, a tool for behavioural pain assessment: translation and pilot-validation in nursing home patients with cognitive impairment. Palliat Med.

[33] Parmelee PA, Smith B, Katz IR (1993). Pain complaints and cognitive status among elderly institution residents. J Am Geriatr Soc.

[34] Weiner D, Peterson B, Keefe F (1998). Evaluating persistent pain in long term care residents: what role for pain maps?. Pain.

[35] Abbey J, Piller N, De Bellis A, Esterman A, Parker D, Giles L, Lowcay B (2004). The Abbey pain scale: a 1-minute numerical indicator for people with end-stage dementia. Int J Palliat Nurs.

[36] Takai Y, Yamamoto-Mitani N, Chiba Y, Nishikawa Y, Hayashi K, Sugai Y (2010). Abbey Pain Scale: development and validation of the Japanese version. Geriatr Gerontol Int.

[37] van Iersel T, Timmerman D, Mullie A (2006). Introduction of a pain scale for palliative care patients with cognitive impairment. Int J Palliat Nurs.

[38] Babicova I, Cross A, Forman D, Hughes J, Hoti K (2021). Evaluation of the psychometric properties of PainChek® in UK aged care residents with advanced dementia. BMC Geriatr.

[39] Atee M, Hoti K, Parsons R, Hughes JD (2017). Pain assessment in dementia: evaluation of a point-of-care technological solution. J Alzheimers Dis.

[40] Liu JY, Briggs M, Closs SJ (2010). The psychometric qualities of four observational pain tools (OPTs) for the assessment of pain in elderly people with osteoarthritic pain. J Pain Symptom Manage.

[41] Nygaard HA, Jarland M (2006). The checklist of nonverbal pain indicators (CNPI): testing of reliability and validity in Norwegian nursing homes. Age Ageing.

[42] Ersek M, Polissar N, Neradilek MB (2011). Development of a composite pain measure for persons with advanced dementia: exploratory analyses in self-reporting nursing home residents. J Pain Symptom Manage.

[43] Neville C, Ostini R (2014). A psychometric evaluation of three pain rating scales for people with moderate to severe dementia. Pain Manag Nurs.

[44] Ersek M, Herr K, Neradilek MB, Buck HG, Black B (2010). Comparing the psychometric properties of the checklist of nonverbal pain Behaviors (CNPI) and the pain assessment in advanced dementia (PAIN-AD) instruments. Pain Med.

[45] Leong IY, Chong MS, Gibson SJ (2006). The use of a self-reported pain measure, a nurse-reported pain measure and the PAINAD in nursing home residents with moderate and severe dementia: a validation study. Age Ageing.

[46] Zwakhalen SM, Hamers JP, Berger MP (2006). The psychometric quality and clinical usefulness of three pain assessment tools for elderly people with dementia. Pain.

[47] Lin PC, Lin LC, Shyu YI, Hua MS (2010). Chinese version of the pain assessment in advanced dementia scale: initial psychometric evaluation. J Adv Nurs.

[48] Torvik K, Kaasa S, Kirkevold O, Rustøen T (2010). Pain and quality of life among residents of Norwegian nursing homes. Pain Manag Nurs.

[49] Chen YH, Lin LC, Watson R (2010). Validating nurses’ and nursing assistants’ report of assessing pain in older people with dementia. J Clin Nurs.

[50] Akbarzadeh M, Jakobsson U (2007). Assessing pain mong older people with communication difficulties—a psychometric evaluation of DOLOPLUS-2. Vard I Norden.

[51] Chen YH, Lin LC, Watson R (2010). Evaluation of the psychometric properties and the clinical feasibility of a Chinese version of the Doloplus-2 scale among cognitively impaired older people with communication difficulty. Int J Nurs Stud.

[52] Fuchs-Lacelle S, Hadjistavropoulos T (2004). Development and preliminary validation of the pain assessment checklist for seniors with limited ability to communicate (PACSLAC). Pain Manag Nurs.

[53] Cheung G, Choi P (2008). The use of the pain assessment checklist for seniors with limited ability to communicate (PACSLAC) by caregivers in dementia care. N Z Med J.

[54] Kaasalainen S, Akhtar-Danesh N, Hadjistavropoulos T, Zwakhalen S, Verreault R (2013). A comparison between behavioral and verbal report pain assessment tools for use with residents in long term care. Pain Manag Nurs.

[55] Chan S, Hadjistavropoulos T, Williams J, Lints-Martindale A (2014). Evidence-based development and initial validation of the pain assessment checklist for seniors with limited ability to communicate-II (PACSLAC-II). Clin J Pain.

[56] Husebo BS, Strand LI, Moe-Nilssen R, Husebo SB, Snow AL, Ljunggren AE (2007). Mobilization-observation-behavior-intensity-dementia pain scale (MOBID): development and validation of a nurse-administered pain assessment tool for use in dementia. J Pain Symptom Manage.

[57] Husebo BS, Strand LI, Moe-Nilssen R, Husebo SB, Ljunggren AE (2010). Pain in older persons with severe dementia. Psychometric properties of the mobilization-observation-behaviour-intensity-dementia (MOBID-2) pain scale in a clinical setting. Scand J Caring Sci.

[58] Husebo BS, Strand LI, Moe-Nilssen R, Husebo SB, Ljunggren AE (2009). Pain behavior and pain intensity in older persons with severe dementia: reliability of the MOBID Pain Scale by video uptake. Scand J Caring Sci.

[59] Husebo BS, Ostelo R, Strand LI (2014). The MOBID-2 pain scale: reliability and responsiveness to pain in patients with dementia. Eur J Pain.

[60] Herr K, Sefcik JS, Neradilek MB, Hilgeman MM, Nash P, Ersek M (2019). Psychometric evaluation of the MOBID dementia pain scale in U.S. nursing homes. Pain Manag Nurs.

[61] Sefcik J, Herr K, Neradilek M, Hilgeman M, Nash P, Ersek M (2018). Psychometrics of the mobility observation-behavior-intensity-dementia (MOBID) pain scale in US nursing homes. Innov Aging.

[62] Zare M, Tagharrobi Z, Sharifi K, Sooki Z, Abolhasani J (2020). Psychometric evaluation of the Persian version of the Doloplus-2 (P-Doloplus-2) scale in elderly with dementia. Turk J Med Sci.

[63] Hølen JC, Saltvedt I, Fayers PM, Hjermstad MJ, Loge JH, Kaasa S (2007). Doloplus-2, a valid tool for behavioural pain assessment?. BMC Geriatr.

[64] Lautenbacher S, Walz AL, Kunz M (2018). Using observational facial descriptors to infer pain in persons with and without dementia. BMC Geriatr.

[65] Cantón-Habas V, Rich-Ruiz M, Moreno-Casbas MT, Ramírez-Expósito MJ, Martínez-Martos JM, Carrera-González MDP (2021). Correlation between biomarkers of pain in saliva and PAINAD scale in elderly people with cognitive impairment and inability to communicate. J Clin Med.

[66] Lorenzet IC, Dos Santos FC, De Souza PMR, Gambarro RC (2011). Assessment of pain in elderly patients with dementia: translation and transcultural adaptation of the instrument PACSLAC into Portuguese. Rev Bras Med.

[67] Bullock L, Chew-Graham CA, Bedson J, Bartlam B, Campbell P (2020). The challenge of pain identification, assessment, and management in people with dementia: a qualitative study. BJGP Open.

[68] Liu JY (2014). Exploring nursing assistants’ roles in the process of pain management for cognitively impaired nursing home residents: a qualitative study. J Adv Nurs.

[69] Bullock L, Bedson J, Jordan JL, Bartlam B, Chew-Graham CA, Campbell P (2019). Pain assessment and pain treatment for community-dwelling people with dementia: a systematic review and narrative synthesis. Int J Geriatr Psychiatry.

[70] Gilmore-Bykovskyi AL, Bowers BJ (2013). Understanding nurses' decisions to treat pain in nursing home residents with dementia. Res Gerontol Nurs.

[71] Whybrow P, Moffatt S, Kay L, Thompson B, Aspray T, Duncan R (2018). Assessing the need for arthritis training among paid carers in UK residential care homes: a focus group and interview study. Musculoskeletal Care.

[72] Hunt LJ, Covinsky KE, Yaffe K (2015). Pain in community-dwelling older adults with dementia: results from the National Health and Aging Trends Study. J Am Geriatr Soc.

[73] Achterberg WP, Erdal A, Husebo BS, Kunz M, Lautenbacher S (2021). Are chronic pain patients with dementia being undermedicated?. J Pain Res.

[74] Roitto HM, Kautiainen H, Aalto UL, Öhman H, Laurila J, Pitkälä KH (2019). Fourteen-year trends in the use of psychotropic medications, opioids, and other sedatives among institutionalized older people in Helsinki. Finland J Am Med Dir Assoc.

