# Supplementary material for: Psychometric properties of pain measurements for people living with dementia: a COSMIN systematic review
Source: Eur Geriatr Med. 2022 May 27;13(5):1029–45. doi: 10.1007/s41999-022-00655-z (PMC9553783; doi:10.1007/s41999-022-00655-z)
Supplement: Supplementary file 1 — Supplementary file1 (DOCX 38 KB) [file 41999_2022_655_MOESM1_ESM.docx]

**Supplementary File 1**: Search strategy (EMBASE example – optimised for other databases)

1. exp Pain/
2. exp Pain Threshold/
3. exp Pain Perception/
4. exp Myalgia/
5. exp Neuralgia/
6. exp Acute Pain/
7. exp Chronic Pain/
8. exp Hyperalgesia/
9. exp Neuritis/
10. exp Paresthesia/
11. (pain or discomfort or allodynia, or neuritis or neuropathy or myalgia or neuralgia or hyperalgesia or paresthesia or soreness or ache* or dys?sthesia or Nocicepti*).ti,ab
12. OR/1-11
13. (aged or elder* or seniors or (old* adj2 (people or person* or patient* or men or women))).mp.
14. exp dementia/
15. exp Alzheimer Disease/
16. exp Cognition Disorders/
17. cognitive impairment.mp.
18. Cognitive function*.mp.
19. (alzheimer* or dement* or "Frontotemporal lobar degeneration" or "Frontotemporal dement*" or Huntington or "Lewy Body disease").tw.
20. OR/14-19
21. AND/13,20
22. exp Pain Measurement/
23. exp psychometrics/
24. exp Symptom Assessment/
25. exp Self Report/
26. (assessment or self report or identification or recognition or detection or evaluation or appraisal or rating).ti,ab
27. (tool* or test*).ti,ab
28. (instrumentation or "validation studies" or "comparative study" or psychometr*[tiab] or clinimetr* or clinometr* or outcome assessment (health care)).ti,ab
29. (“outcome assessment" or "outcome measure*" or "observer variation" or "observer variation" or "health status indicators" or "reproducibility of results" or reproducib* or "discriminant analysis").ti,ab
30. (reliab* or unreliab* or valid* or "coefficient of variation" or coefficient or "internal consistency" or (cronbach* AND (alpha or alphas)) or (item and (correlation* or selection* or reduction*).ti,ab
31. (agreement or precision or imprecision or "precise values").tw
32. (test-retest or (test and retest) or (reliab* and (test or retest)).ti,ab
33. (interrater or inter-rater or intrarater or intra-rater or intertester or inter-tester or intratester or intra-tester or interobserver or inter-observer or intraobserver or intra-observer or intertechnician or inter-technician or intratechnician or intra-technician or interexaminer or inter-examiner or intraexaminer or intra-examiner or interindividual or inter-individual or intraindividual or intra-individual or interparticipant or inter-participant or intraparticipant or intra-participant).ti,ab
34. (Kappa or kappa's or kappas).ti,ab
35. (repeatab* or ((replicab* or repeated) and (measure or measures or findings or result or results or test or tests)).ti,ab
36. (generaliza* or generalisa* or concordance or (intraclass and correlation*) or discriminative or "known group" or "factor analysis" or "factor analyses" or "factor structure" or "factor structures" or dimension*).ti,ab
37. (subscale* or (multitrait and scaling and (analysis or analyses)) or "item discriminant" or "interscale correlation*" or error or errors or "individual variability" or "interval variability" or "rate variability" or (variability AND (analysis OR values)).ti,ab
38. (uncertainty and (measurement or measuring)) or "standard error of measurement" or sensitiv*).t,ab
39. (responsive* or (limit and detection) or "minimal detectable concentration" or interpretab* or ((minimal or minimally or clinical or clinically) and (important or significant or detectable) and (change or difference)) or (small* and (real or detectable) and (change or difference)) or "meaningful change").ti,ab
40. (“ceiling effect" or "floor effect").ti,ab
41. ("Item response model" or IRT or Rasch or "Differential item functioning" or DIF or "computer adaptive testing" or "item bank"or "cross-cultural equivalence").ti,ab
42. OR/18-37
43. AND/12,21,38

**Supplementary File 2:** The included pain assessment instruments examined with their original reference.

| **Pain Assessment Instrument** | **Original Citation** |
| --- | --- |
| Abbey Pain Scale | Abbey J, Piller N, De Bellis A, Esterman A, Parker D, Giles L, Lowcay B. The Abbey pain scale: a 1-minute numerical indicator for people with end-stage dementia. Int J Palliat Nurs. 2004;10:6-13. |
| ALGOPLUS | Rat P, Jouve E, Pickering G, Donnarel L, Nguyen L, Michel M, Capriz-Ribière F, Lefebvre-Chapiro S, Gauquelin F, Bonin-Guillaume S. Validation of an acute pain-behavior scale for older persons with inability to communicate verbally: Algoplus. Eur J Pain. 2011;15:198.e1-198.e10 |
| Checklist for non-verbal pain behavior (CNPI) | Feldt KS. The checklist of nonverbal pain indicators (CNPI). Pain Manag Nurs. 2000;1:13-21. |
| DOLOPLUS-2 | Wary B, collectief Doloplus: Doloplus-2, une échelle pour évaluer la douleur. Soins Gérontologie. 1999;19:25-7.  Lefebre-Chapiro L, Doloplus group: The Doloplus 2 scale-evaluating pain in the elderly. European Journal of Palliative Care. 2001;8:191-4. |
| Facial Action Coding System | Ekman P, Friesen W. Facial Action Coding System: a technique for the measurement of facial movement. Consulting Psychologists Press, Palo Alto; 1978. |
| MOBID-2 | Husebo BS, Strand LI, Moe-Nilssen R, Husebo SB, Ljunggren AE. Pain in older persons with severe dementia. Psychometric properties of the Mobilization-Observation-Behaviour-Intensity-Dementia (MOBID-2) Pain Scale in a clinical setting. Scand J Caring Sci. 2010;24:380-91. |
| Pain Assessment in Advanced Dementia (PAINAD) | Warden V, Hurley AC, Volicer L. Development and psychometric evaluation of the Pain Assessment in Advanced Dementia (PAINAD) scale. J Am Med Dir Assoc. 2003;4:9-15. |
| PACSLAC/PACSLAC-2 | Fuchs-Lacelle S, Hadjistavropoulos HD: Development and preliminary validation of the Pain Assessment Checklist for Seniors with Limited Ability to Communicate (PACSLAC). Pain Management Nursing. 2004;1:37-49.  Chan S, Hadjistavropoulos T, Williams J, Lints-Martindale A. Evidence-based development and initial validation of the pain assessment checklist for seniors with limited ability to communicate-II (PACSLAC-II). Clin J Pain. 2014;30:816-24. |
| Self-reported pain through the NRS or VAS/thermometer or Philadelphia Geriatric Pain Intensity Scale | Parmelee PA, Smith B, Katz I. Pain complaints and cognitive status among elderly institution residents. J Am Geriatr Soc. 1993;41:517-22. |

**Supplementary File 3**: Results of the COSMIN methodological quality assessment for each included study

| **Study** | **PROM Development** | **Internal consistency** | **Reliability** | **Measure error** | **Construct Validity** | **Structural validity** | **Hypothesetesting** | **Cross-cultural validity/** | **Criterion validity** | **Responsiveness** | **Interpret.** |
| --- | --- | --- | --- | --- | --- | --- | --- | --- | --- | --- | --- |
| Abbey [35] |  |  |  |  |  |  |  |  |  |  |  |
| Akbarzadeh [50] |  |  |  |  |  |  |  |  |  |  |  |
| Ando [25] |  |  |  |  |  |  |  |  |  |  |  |
| Ando [26] |  |  |  |  |  |  |  |  |  |  |  |
| Atee [39] |  |  |  |  |  |  |  |  |  |  |  |
| Babicova [38] |  |  |  |  |  |  |  |  |  |  |  |
| Batalha [21] |  |  |  |  |  |  |  |  |  |  |  |
| Browne [30] |  |  |  |  |  |  |  |  |  |  |  |
| Büyükturan [16] |  |  |  |  |  |  |  |  |  |  |  |
| Cantón-Habas [24] |  |  |  |  |  |  |  |  |  |  |  |
| Cantón-Habas [65] |  |  |  |  |  |  |  |  |  |  |  |
| Chan [55] |  |  |  |  |  |  |  |  |  |  |  |
| Chen [49] |  |  |  |  |  |  |  |  |  |  |  |
| Chen [51] |  |  |  |  |  |  |  |  |  |  |  |
| Cheung [53] |  |  |  |  |  |  |  |  |  |  |  |
| Costardi [20] |  |  |  |  |  |  |  |  |  |  |  |
| Ersek [44] |  |  |  |  |  |  |  |  |  |  |  |
| Ersek [42] |  |  |  |  |  |  |  |  |  |  |  |
| Feldt [19] |  |  |  |  |  |  |  |  |  |  |  |
| Fuchs-Lacelle [52] |  |  |  |  |  |  |  |  |  |  |  |
| Hadjistavropoulos [31] |  |  |  |  |  |  |  |  |  |  |  |
| Herr [60] |  |  |  |  |  |  |  |  |  |  |  |
| Holen [32] |  |  |  |  |  |  |  |  |  |  |  |
| Holen [63] |  |  |  |  |  |  |  |  |  |  |  |
| Husebo [56] |  |  |  |  |  |  |  |  |  |  |  |
| Husebo [58] |  |  |  |  |  |  |  |  |  |  |  |
| Husebo [57] |  |  |  |  |  |  |  |  |  |  |  |
| Husebo [59] |  |  |  |  |  |  |  |  |  |  |  |
| Kaasalainen [54] |  |  |  |  |  |  |  |  |  |  |  |
| Kunz [18] |  |  |  |  |  |  |  |  |  |  |  |
| Lautenbacher [64] |  |  |  |  |  |  |  |  |  |  |  |
| Leong [45] |  |  |  |  |  |  |  |  |  |  |  |
| Lin [47] |  |  |  |  |  |  |  |  |  |  |  |
| Liu [40] |  |  |  |  |  |  |  |  |  |  |  |
| Lorenzet [66] |  |  |  |  |  |  |  |  |  |  |  |
| Neville [43] |  |  |  |  |  |  |  |  |  |  |  |
| Nygaard [41] |  |  |  |  |  |  |  |  |  |  |  |
| Parmelee [33] |  |  |  |  |  |  |  |  |  |  |  |
| Pateux [28] |  |  |  |  |  |  |  |  |  |  |  |
| Pautex [27] |  |  |  |  |  |  |  |  |  |  |  |
| Pinto [23] |  |  |  |  |  |  |  |  |  |  |  |
| Rat [29] |  |  |  |  |  |  |  |  |  |  |  |
| Sefcik [61] |  |  |  |  |  |  |  |  |  |  |  |
| Takai [36] |  |  |  |  |  |  |  |  |  |  |  |
| Thé [17] |  |  |  |  |  |  |  |  |  |  |  |
| Torvik [48] |  |  |  |  |  |  |  |  |  |  |  |
| Valera [22] |  |  |  |  |  |  |  |  |  |  |  |
| Van Iersel [37] |  |  |  |  |  |  |  |  |  |  |  |
| Weiner [34] |  |  |  |  |  |  |  |  |  |  |  |
| Zare [62] |  |  |  |  |  |  |  |  |  |  |  |
| Zwakhalen [46] |  |  |  |  |  |  |  |  |  |  |  |

**Rating:** VG - Very good; adequate; inadequate; doubtful; not reported

**Supplementary File 4:** Psychometric results extracted by study

|  | **Construct validity** | **Structural validity** | **Reliability (ICC)** | **Internal consistency (Cronbach)** | **Responsiveness (to treatment or rest)** | **Measurement error** |
| --- | --- | --- | --- | --- | --- | --- |
| Abbey [35] | R=0.586; P<0.001 |  |  | 0.74 | P<0.001 |  |
| Akbarzadeh [50] | R=0.698; P=<0.001 to UAB | 36.9% variance explained | Inter: 0.90 |  |  |  |
| Ando [25] |  |  | Inter: 0.90 |  |  |  |
| Ando [26] |  |  |  |  | P<0.001 |  |
| Atee [39] | R=0.91 with PainChek (p<0.001) |  | Inter: 0.86  Intra: 0.90 | 0.95 | p<0.001 |  |
| Babicova [38] | R=0.82 with PainChek (p<0.001) |  | Inter: 0.72 intra: 0.68 | 0.81 | p<0.001 |  |
| Batalha [21] |  | 61.1% variance explain | Intra:0.89 | 0.84 |  |  |
| Browne [30] |  |  | Inter: 0.94  Inter: 0.86 |  |  |  |
| Büyükturan [16] | CVI: 0.84  P<0.001 to nurse VAS | 68.9% variance explained | Intra: 0.81 |  |  |  |
| Cantón-Habas [24] | CVI: 0.875  P<0.02 to medication use | 62.5% variance explained | Inter: 0.94  Intra: 0.80-0.83 | 0.76 |  |  |
| Cantón-Habas [65] | P<0.01 to sTNF-RII and sIgA pain biomarkers | 46.5% variance explained |  |  |  |  |
| Chan [55] | R=0.68; p<0.01 to PACSLAC-II; p<0.01 to CNPI; R=0.79; p<0.01 to PACSLAC-II |  | Inter: 0.75-0.97 intra: 0.88-0.90  Inter: 0.63 | 0.69-0.80  0.74-0.77 | p<0.01 |  |
| Chen [49] |  | 70.4% variance explained | Inter: 0.35 |  |  |  |
| Chen [51] |  | 65% variance explained | Inter: 0.81 | 0.74 |  |  |
| Cheung [53] |  |  | Inter: R=0.83 |  |  |  |
| Costardi [20] | R=0.65; p=0.008 |  | Inter: R=0.87  Intra: R=0.88 | 0.74 |  |  |
| Ersek [44] | R=0.48; p<0.001; R=0.41; P<0.05 |  | Intra:0.80  Inter:0.04  Intra:0.65  Inter:0.25 | 0.72; 0.90 |  |  |
| Ersek [42] | P<0.001 |  |  |  |  |  |
| Feldt [19] | R=0.46 (p<0.01) |  | Intra: 0.60  Inter: 0.86-0.90 | 0.64 |  |  |
| Fuchs-Lacelle [52] | R=0.54; p<0.001 to global intensity rating |  |  | 0.82-0.87 |  |  |
| Hadjistavropoulos [31] | R=0.542; p<0.01; R=0.463; p<0.01 |  | Inter: R=0.76; Inter: R=0.89 |  |  |  |
| Herr [60] | R=0.54 P<0.001 to caregiver NRS |  |  | 0.83 |  |  |
| Holen [32] |  | 68% variance explained |  |  |  |  |
| Holen [63] | R2=0.023 |  | Intra: 0.74  Inter: 0.77 |  |  |  |
| Husebo [56] |  |  | Inter: 0.86 | 0.86-0.90 |  |  |
| Husebo [58] |  |  | Intra: 0.79-0.92  Inter: 0.86-0.97 |  |  |  |
| Husebo [57] | R2=0.61; P<0.01 to caregiver NRS |  | Inter: 0.94  Intra: 0.92 | 0.82-0.84 |  |  |
| Husebo [59] |  |  | Intra: 0.852 |  | p<0.001  MCID: 3 points | SEM: 1.4 |
| Kaasalainen [54] | p<0.01 with NRS; p<0.01 with PACSLAC |  | Inter: 0.87 |  | p<0.001; p=0.03 |  |
| Kunz [18] | P<0.001 |  |  |  |  |  |
| Lautenbacher [64] | R^2^=0.116 P<0.001 | P=0.006 to p<0.001 |  |  |  |  |
| Leong [45] | R=0.304; p<0.01; R=0.842; p<0.001 |  |  |  |  |  |
| Lin [47] |  | 62.5 variance explained | Intra: 0.71  Inter: 0.84 | 0.55 | P<0.001 |  |
| Liu [40] | P<0.01 | Cronbach: 0.75  Cronbach: 0.76 | Inter: 0.90  Inter: 0.88  Inter: 0.82 | 0.73  0.72  0.77 |  |  |
| Neville [43] | P<0.01 |  | Intra:0.68  Inter:0.75  Intra:0.71  Inter:0.73  Intra:0.56  Inter:0.59 | 0.74; 0.76; 0.86 |  |  |
| Nygaard [41] | R=0.88 (p<0.001) |  |  |  |  |  |
| Parmelee [33] |  |  | Intra: 0.84 | 0.84 |  |  |
| Pateux [27] | R2=0.46 P<0.01 to DOLOPLUS; R2=0.46 P<0.01 to VAS self-assess |  |  |  |  |  |
| Pateux [28] |  |  | Inter: 0.97  Intra: 0.71-0.80 |  |  |  |
| Pinto [23] |  |  | Inter: 0.79 | 0.65 |  |  |
| Rat [29] | R2=0.81 (p<0.001) to VAS Pain; R2=0.81 (p<0.001) to Algoplus |  | Inter: 0.812; Inter: 0.43-0.80 | KR-20: 0.712 | P<0.001 |  |
| Sefcik [61] | R2=0.51 P<0.001 to caregiver NRS |  |  | 0.83 |  |  |
| Thé [17] | R=0.64; P<0.001 to caregiver VAS |  | Inter: 0.85  Inter: 0.64 | 0.827 |  |  |
| Takai [36] | R=0.49 P<0.01 |  | Inter: 0.82  Intra: 0.66 | 0.65 |  |  |
| Torvik [48] | P=0.01 agree to nursing VAS |  |  | 0.71 |  |  |
| Weiner [34] | R=0.95 |  | Intra: 0.71-0.85 |  |  |  |
| Zare [62] |  | 76.14% variance explained | Inter: 0.86 | 0.950 |  | SEM: ± 1.759 |
| Zwakhalen [46] | R=0.81 to VAS nurse (p<0.01) |  | Inter: 0.81  Intra: 0.89 | 0.72 |  |  |
| Zwakhalen [46] | R=0.72 to VAS nurse (p<0.01) |  | Inter: 0.96  Intra: 0.86 | 0.84 |  |  |
| Zwakhalen [46] | R=0.33 to VAS nurse (p<0.01) |  | Inter: 0.78 intra:0.85 | 0.74 |  |  |

CVI: Content Validity Index; ICC – Intra-class correlation coefficient; MCID – minimally clinical important difference; NRS – numerical rating scale; SEM – standard error of mean; VAS – visual analogue scale
